# Transparent peer review at *Nature Communications*

**DOI:** 10.1038/ncomms10277

**Published:** 2015-12-14

## Abstract

Authors of papers submitted from January 2016 will be given the option to publish the peer review history of their paper

Scientific peer review is regularly criticized as being ineffective and ‘broken', although it continues to be widely used in academic publishing. While peer review certainly has its drawbacks, we regard this process as an essential valuable mechanism to improve the quality and accuracy of the scientific record, and it helps us editors to decide about the publication of a paper. Reviewer reports provide fascinating insights into the merits and limitations of our papers and often foster revisions that make a substantial difference to the robustness and impact of the papers that we publish. A study that challenges established knowledge can lead to long, deep discussions between authors, editors and reviewers that so far, at our journal, remain confidential.

As a journal, we are committed to openness and transparency, and to make our peer review process more transparent, as a trial, we will be publishing all reviewer comments to authors and author rebuttal letters for published papers submitted from January 2016, unless the authors ask us not to. Upon acceptance of a paper, authors will be asked whether they agree to the publication of the peer review file along with the accepted version of the manuscript. We will evaluate the success of the trial by monitoring parameters such as author opt-out rates, and are planning to provide a report within a year.

**By enabling the publication of these peer review documents, we take a step towards opening up our editorial decision-making process.**

These files will only contain the peer review history at *Nature Communications*. If a manuscript is submitted to us after review at another *Nature* journal, those referee reports and author rebuttal letters will remain confidential, as those reports were submitted without prior consent to be published. Similarly, peer review identities will only be released if a reviewer explicitly agrees to this, for example by signing the comments made to the authors with their name. Apart from such circumstances, we will continue to honour reviewer anonymity. Further details can be found in a separate FAQ document at http://www.nature.com/ncomms/authors/ncomms-transparent-peer-review.pdf.

As a multidisciplinary journal, transparent peer review might be new to some of our authors, but we are not the first journal to implement this process. Similar schemes have already been successfully implemented in various scientific disciplines by the European Geosciences Union, at BioMed Central, for EMBO journals, and elsewhere.

By enabling the publication of these peer review documents, we take a step towards opening up our editorial decision-making process. We hope that this will contribute to the scientific appreciation of the papers that we publish, in the same way as these results are already discussed at scientific conferences. We also hope that publication of the reviewer reports provides more credit to the work of our reviewers, as their assessment of a paper will now reach a much larger audience.

It is important to note that published peer review files will focus on the comments of the reviewers and the authors' responses to them. Correspondence between authors and editors, reviewer comments only to the editors, as well as the internal discussions between editors will remain confidential. In doing so, we aim to strike a balance between ensuring that the information that led to the decision to publish the paper is freely available, and at the same time enabling reviewers, authors and editors to communicate frankly and privately where necessary. In cases where not all of the circumstances that lead to an editorial decision on a manuscript are fully captured in peer review files, editorial comments may be added to the peer review document. The ultimate decision whether to publish a paper lies with the editors, and is always taken after careful consideration of all arguments raised.

This move towards more transparency will coincide with the completion of our transformation to full open access. From January all new papers will be published with a Creative Commons open access licence, and all subscription-based content from our archive will be made free to read and is available for non-commerical text and data mining. We hope that our readers and authors will enjoy the free access to our papers and the forthcoming peer review files, and that this will support and enhance the understanding and accessibility of our published papers.

